# The impact of a multi-micronutrient nutritional formula combined with cognitive behavioral therapy in managing symptoms of children with attention deficit hyperactivity disorder

**DOI:** 10.3389/fped.2025.1624344

**Published:** 2025-07-07

**Authors:** Yun Wang, Meng Cao

**Affiliations:** ^1^ Department of Pediatrics, Wuxi No. 2 People’s Hospital, Wuxi, Jiangsu, China; ^2^ Department of Endocrinology, The First Affiliated Hospital of Xinxiang Medical University, Xinxiang, Henan, China

**Keywords:** ADHD, cognitive behavioral therapy, multi-Micronutrient supplementation, pediatric psychology, attention-deficit/hyperactivity disorder

## Abstract

**Background:**

Attention-Deficit/Hyperactivity Disorder (ADHD) characterized by symptoms of inattention, hyperactivity, and impulsivity, with substantial impacts on a child's academic performance, social interactions, and overall quality of life. This study investigates the impact of combining a multi-micronutrient nutritional formula (MNF) with cognitive behavioral therapy (CBT) in managing ADHD symptoms in children.

**Methods:**

In a retrospective analysis of 220 children aged 6–14 years diagnosed with ADHD, patients were divided into two groups: the MNF group (*n* = 112) receiving only the nutritional formula, and the MNF-CBT group (*n* = 108) receiving both the nutritional formula and CBT. The interventions were conducted over three months. Outcomes were measured using the Conners Parent Symptom Questionnaire (PSQ), SNAP-IV Teacher Form, and Dundee Difficult Times of the Day Scale (D-DTODS).

**Results:**

Both groups were comparable at baseline in terms of demographics and ADHD symptom severity. Post-treatment analyses revealed greater reductions in attention deficit scores, hyperactivity/impulsivity scores, and oppositional symptoms scores in the MNF-CBT group compared to the MNF group. Conduct scores were lower in the MNF-CBT group than in the MNF group, and learning scores also showed a similar pattern of reduction. Functional impairments across various daily activities, as measured by the D-DTODS, were also reduced in the MNF-CBT group. No adverse events or safety concerns were observed during the intervention period.

**Conclusion:**

The combination of multi-micronutrient supplementation with CBT shows potential in alleviating both neurochemical deficiencies and behavioral dysregulation in children with ADHD. These findings suggest that this integrated approach may be beneficial for managing ADHD symptoms.

## 1 Introduction

Attention-Deficit/Hyperactivity Disorder (ADHD) was a prevalent neurodevelopmental disorder characterized by symptoms of inattention, hyperactivity, and impulsivity, often resulting in substantial impairments in social, academic, and occupational functioning (1, 2). In China, the age-standardized prevalence of ADHD increased by 9.86% from 1990–2021 (3). Over the past few decades, traditional pharmacological interventions for ADHD, primarily stimulant medications, have demonstrated impact in reducing core symptoms (4, 5). However, these treatments often come with limitations, including potential side effects, and do not typically address the broader spectrum of cognitive challenges faced by children with ADHD (6, 7). Additionally, there was increasing recognition of the role those nutritional deficiencies, particularly in micronutrients, may play in the pathophysiology of ADHD. This recognition has spurred interest in complementary non-pharmacological treatments, such as nutritional interventions, which may provide a beneficial adjunct or alternative to medication (8, 9).

Micronutrients, essential for maintaining optimal brain function, were critical in various neurobiological processes, including neurotransmitter synthesis, synaptic plasticity, and neuroprotection. Previous studies have highlighted the potential of certain micronutrients, such as zinc, magnesium, iron, and B-complex vitamins, in modulating neurodevelopmental. Nutritional deficits, particularly in essential vitamins and minerals like zinc, magnesium, iron, and B-complex vitamins, may exacerbate ADHD symptoms due to their critical involvement in neurotransmitter synthesis, neuronal growth, and cognitive function (10, 11). Therefore, a multi-micronutrient nutritional formula (MNF) could potentially rectify these deficiencies and alleviate symptoms by restoring biochemical balance and enhancing overall neurological health.

Simultaneously, non-pharmacological therapies such as Cognitive Behavioral Therapy (CBT) have gained traction as effective modalities for managing ADHD, particularly in addressing specific psychosocial challenges and behavioral patterns (12, 13). CBT was an established psychological intervention with demonstrated impact in modifying patterns and behaviors. CBT focuses on modifying dysfunctional thought processes and developing coping strategies, thus offering symptomatic improvement and better functional outcomes when integrated into a child's treatment plan. By focusing on cognitive restructuring and emotion regulation, CBT equips individuals with strategies to manage symptoms effectively and improve functional outcomes. When combined with nutritional support, CBT holds promise for creating synergistic effects, potentially through mechanisms such as enhanced neurotransmitter synthesis and improved cognitive function due to the complementary action of MNF in supporting neural pathways and CBT in improving behavioral control. This presents a compelling rationale for evaluating the combined therapeutic potential of MNF and CBT in children facing the dual challenge of ADHD (14, 15). Other non-pharmacological interventions, including parent training programs, school-based interventions, and social skills training, also play important roles in managing ADHD symptoms. These approaches complement both pharmacological and nutritional interventions by addressing broader psychosocial needs.

Historically, the interplay between nutrition and neurodevelopment has been underexplored in clinical research. In this context, our study seeks to explore the impact of a combined intervention using MNF and CBT in managing symptoms of children with ADHD. We hypothesize that the combination of MNF and CBT will result in greater improvements in ADHD symptoms compared to either intervention alone.

## 2 Materials and methods

### 2.1 Participant eligibility criteria

Participants qualified for the study if they fulfilled the following criteria: having a diagnosis of ADHD in accordance with the American Psychiatric Association (APA) guidelines (16); aged between 6 and 14 years, since children under 6 were excluded due to challenges in engaging them in the intervention, adolescents older than 14 years were excluded because of the more complex physical and psychological development, which could potentially confound the study results; and having complete medical records available.

Exclusion criteria included use of asthma or allergic rhinitis medications; use of other medications or supplements that could affect ADHD symptoms; allergy to the trace elements in the study's dietary supplements; concurrent receipt of other types or forms of behavioral therapy or psychological treatment; severe physical illness or disability; intellectual disability; and a history of brain injury; prior treatments for ADHD within the past six months; and significant parental involvement in the treatment regimen.

Approval for this study was obtained from the Institutional Review Board (IRB) and Ethics Committee at WuXi No. 2 People's Hospital. Since the study involved only de-identified patient data, which posed no direct risk or impact on the patients, the IRB and Ethics Committee granted a waiver for informed consent, in line with the regulatory and ethical standards governing retrospective research.

### 2.2 Research design and cohort segmentation

A retrospective analysis was conducted on 220 children diagnosed with ADHD, who received treatment at WuXi No. 2 People's Hospital between November 2022 and October 2024. The participants were categorized into two groups based on their treatment regimen. Patients who were treated solely with the MNF were defined as the MNF group, while those who received both MNF and CBT were categorized as the MNF + CBT group. The assignment to each treatment regimen was based on the availability of CBT resources during the enrollment period. Specifically, patients in the MNF group accessed care when CBT services were not available, whereas those in the MNF + CBT group were treated during periods when CBT services were accessible. This approach aimed to minimize potential selection bias by ensuring that access to CBT was driven by service availability rather than clinical judgment or parental preference.

### 2.3 Intervention protocols and monitoring

The MNF group received a three-month regimen of a standardized multi-micronutrient formula containing essential nutrients with adjusted dosages appropriate for children aged 6–14 years: Zinc 8 mg/day, Magnesium 150 mg/day, Iron 8 mg/day for both males and females, Vitamin B1 (Thiamine) 0.9 mg/day, Vitamin B2 (Riboflavin) 1.1 mg/day, Vitamin B3 (Niacin) 12 mg/day, Vitamin B6 (Pyridoxine) 1.0 mg/day, Vitamin B12 (Cobalamin) 1.8 µg/day, and Folate 200 µg/day. These nutrients were primarily sourced from high-quality supplements provided by DSM Nutritional Products to ensure purity and potency, but also supplemented through carefully monitored natural food sources such as red meat, seafood, legumes, whole grains, nuts, seeds, leafy green vegetables, and fortified foods. Standardization and quality control processes included rigorous testing for each batch to meet the specified nutrient levels, with healthcare professionals supervising the daily dietary intake to ensure all participants received identical meals based on this standardized formulation.

The MNF-CBT group not only received the MNF but also participated in CBT three-months. Each child attended three 60 min CBT sessions per week, following a treatment manual developed by E. Jane Costello and John S. March titled “Cognitive Behavioral Therapy for Children and Adolescents: A Guide for Clinicians” published by Elsevier to ensure standard delivery and adherence. The sessions included several components:

Cognitive Restructuring: Children were taught to identify their immediate reactive thoughts to events, evaluate the validity of these negative thoughts, and develop more positive and realistic perspectives. They kept records of their feelings and thoughts to identify triggers for emotional fluctuations.
Emotion Regulation Techniques: Skills taught included deep breathing, progressive muscle relaxation, and mindfulness practices to reduce anxiety and stress. Creative activities like art and journaling were encouraged to express emotions.
Social Skills Training: This focused on improving effective listening, clear expression, and constructive management of interpersonal conflicts through group activities aimed at enhancing cooperation and empathy.
Family Involvement: Regular meetings with families discussed the child's progress and any arising issues. Parents received guidance on providing better support, setting realistic expectations, and applying consistent discipline methods.
Structured Environment Establishment: A fixed daily routine was implemented, including set times for waking up, eating, homework, and sleeping, to provide stability. Large tasks were divided into smaller steps with rewards for each completed step to boost accomplishment and reduce frustration.

All therapeutic interventions were supervised by licensed mental health professionals who had completed specialized training in CBT for ADHD. All participants in both groups received similar levels of interaction and monitoring, thus controlling for attention-related biases. Fidelity checks were conducted through weekly supervision meetings throughout the intervention period. Adherence to both MNF and CBT interventions was additionally monitored through regular pill counts, dietary logs maintained by parents or guardians, and attendance records for CBT sessions. The SNAP-IV scale was used by instructors to assess outcomes before and after the intervention.

Patient data were collected from the medical record system, including demographic characteristics, baseline disease characteristics and scores from the Conners Parent Symptom Questionnaire (PSQ), Swanson, Nolan, and Pelham-IV (SNAP-IV) Teacher Form, and Dundee Difficult Times of the Day Scale (D-DTODS). Primary Outcome: PSQ scores; Secondary Outcomes: SNAP-IV scores, D-DTODS scores.

### 2.4 Evaluation of ADHD symptomatology

The Conners Parent Symptom Questionnaire (PSQ) was employed to evaluate symptoms of ADHD before and after the intervention. The PSQ measures various domains, including impulsivity and hyperactivity, psychosomatic disorders, learning difficulties, anxiety, conduct problems, and provides a hyperactivity index, encompassing a total of 48 items. Each item was rated on a 4-point scale ranging from 0–3, resulting in a maximum possible score of 144. Higher scores suggest more severe behavioral issues. The PSQ demonstrates strong internal consistency, as indicated by a Cronbach's α of 0.86 (17).

The Chinese version of the SNAP-IV Teacher Form was employed to assess ADHD symptoms and monitor their improvement over time. This scale includes 26 items divided into three domains: inattention (IA) covering items 1–9, hyperactivity/impulsivity (HI) covering items 10–18, and oppositional symptoms (OP) covering items 19–26. Each item was rated on a four-point Likert scale, where 0 indicates “not at all,” 2 signifies “just a little,” 3 corresponds to “quite a bit,” and 4 represents “very much.” The scale demonstrates good internal consistency, with a Cronbach's α coefficient of 0.88 (18).

### 2.5 Assessment of functional impairment

The D-DTODS was utilized to evaluate ADHD-related functional impairments at specific times of the day, based on the average number of days per month. Before and after treatment, parents were asked to assess their children's ADHD-related difficulties at home and school during these periods. The D-DTODS comprises ten items, each rated on a 4-point scale from 1–4, to measure the level of difficulty during the following times: “before school,” “in school,” “after school,” “evening,” and “before bed.” The total score was determined by summing the ten items, with higher scores reflecting greater difficulties in the child's activities. The D-DTODS has a Cronbach's alpha coefficient of 0.793, indicating good internal consistency (19).

### 2.6 Statistical approaches

Statistical analysis was conducted with SPSS Statistics software (version 29.0; IBM Corp., Armonk, NY, USA). Categorical variables were summarized using frequency distributions with proportional compositions, expressed as counts (percentages). Group differences in categorical measures were evaluated through Pearson's chi-square analysis. For continuous variables, distribution normality was verified using the Shapiro–Wilk procedure (*α* = 0.05) to determine appropriate parametric assumptions. Normally distributed metrics were characterized by mean values with standard deviations (M ± SD), while between-group comparisons employed independent samples *t*-tests with equal variance assumptions. All statistical inferences adopted a two-tailed significance threshold of *p* < 0.05.

A power analysis was performed using G*Power software to ensure adequate statistical power for detecting meaningful differences in the primary outcome measure. Based on an assumed medium effect size (Cohen's d = 0.5), a two-tailed significance level (α) of 0.05, and a desired power of 95%, the analysis indicated that each group would require at least 105 participants to detect significant differences in the PSQ Hyperactivity Index using a two-sided, two-sample *t*-test with equal variances. Our current sample size exceeds this requirement, ensuring sufficient statistical power to detect clinically relevant effects.

## 3 Results

### 3.1 Basic data

The mean age of children in the MNF group was 10.38 ± 1.36 years, while in the MNF-CBT group it was 10.46 ± 1.07 years (*t* *=* 0. 487, *P* *=* 0. 627) (Table 1). Body mass index (BMI) measurements were similar, with a mean of 16.91 ± 2.53 kg/m² in the MNF group and 17.24 ± 2.14 kg/m² in the MNF-CBT group (*t* *=* 1.017, *P* *=* 0.310). The distribution of ethnicity (Han vs. Other), sex, and monthly household income per person showed no significant differences between the groups (all *P* > 0.05). The duration of illness averaged 2.76 ± 0.93 years for the MNF group and 2.59 ± 0.89 years for the MNF-CBT group (*t* *=* 1.410, *P* *=* 0.160) (Table 2). Daily medication dosages were also not significantly different between the MNF (27.52 ± 8.78 mg) and MNF-CBT (26.91 ± 8.48 mg) groups (*t* *=* 0.519, *P* *=* 0.604). Allergy history, family history of ADHD, ADHD type also showed no significant differences (all *P* > 0.05). Thus, the baseline characteristics were well-matched, allowing for a valid comparison of the combined treatment's impact.

**Table 1:** Comparison of demographic characteristics between two groups.

Parameters	MNF group (*n* = 112)	MNF-CBT group (*n* = 108)	*t*/*χ*^2^	*P*
Age (years)	10.38 ± 1.36	10.46 ± 1.07	0.487	0.627
BMI (kg/m²)	16.91 ± 2.53	17.24 ± 2.14	1.017	0.310
Ethnicity (Han/Other) [n (%)]	105 (93.75%)/7 (6.25%)	104 (96.3%)/4 (3.7%)	0.750	0.386
Female/Male [n (%)]	51 (45.54%)/61 (54.46%)	46 (42.59%)/62 (57.41%)	0.193	0.660
Monthly household income/person [n (%)]			1.715	0.424
−2,000–5,000	20 (17.86%)	15 (13.89%)		
−5,000–10,000	64 (57.14%)	58 (53.7%)		
-above 10,000	28 (25%)	35 (32.41%)		

BMI, body mass index.

**Table 2:** Comparison of baseline disease characteristics between two groups.

Parameters	MNF group (*n* = 112)	MNF-CBT group (*n* = 108)	*t*/χ^2^	*P*
Duration of illness (year)	2.76 ± 0.93	2.59 ± 0.89	1.410	0.160
Daily medication (mg)	27.52 ± 8.78	26.91 ± 8.48	0.519	0.604
Allergy history [n (%)]	25 (22.32%)	20 (18.52%)	0.489	0.485
Family history of ADHD [n (%)]	15 (13.39%)	12 (11.11%)	0.266	0.606
ADHD type [n (%)]			0.337	0.845
-ADHD-I	30 (26.79%)	28 (25.93%)		
-ADHD-HI	15 (13.39%)	12 (11.11%)		
-ADHD-C	67 (59.82%)	68 (62.96%)		

ADHD, attention deficit hyperactivity disorder; ADHD-I, predominantly inattentive subtype; ADHD-HI, predominantly hyperactive-impulsive subtype; ADHD-C, combined hyperactive-impulsive and inattentive subtype.

### 3.2 ADHD symptom

The conduct scores were 1.96 ± 0.46 for the MNF group and 2.05 ± 0.48 for the MNF-CBT group (*t* *=* 1.475, *P* *=* 0.142), while the learning scores were 1.98 ± 0.38 and 2.06 ± 0.41, respectively (*t* *=* 1.355, *P* *=* 0.177) (Table 3). Psychosomatic disorder scores were slightly different, with 1.99 ± 0.41 in the MNF group and 1.93 ± 0.36 in the MNF-CBT group (*t* *=* 1.258, *P* *=* 0.210). Impulsivity and hyperactivity scores were closely matched at 1.92 ± 0.37 for MNF and 1.89 ± 0.45 for MNF-CBT (*t* *=* 0.475, *P* *=* 0.635). Anxiety scores were also similar between groups, with the MNF group at 1.63 ± 0.42 and the MNF-CBT group at 1.67 ± 0.36 (*t* *=* 0.716, *P* *=* 0.475). Lastly, the hyperactivity index showed no significant difference, with scores of 2.55 ± 0.56 in the MNF group and 2.53 ± 0.57 in the MNF-CBT group (*t* *=* 0.281, *P* *=* 0.779). These findings indicate comparability between groups before initiating treatment interventions.

**Table 3:** Comparison of PSQ score between two groups (before treatment).

Parameters	MNF group (*n* = 112)	MNF-CBT group (*n* = 108)	*t*	*P*
Conduct	1.96 ± 0.46	2.05 ± 0.48	1.475	0.142
Learning	1.98 ± 0.38	2.06 ± 0.41	1.355	0.177
Psychosomatic disorders	1.99 ± 0.41	1.93 ± 0.36	1.258	0.210
Impulsivity and hyperactivity	1.92 ± 0.37	1.89 ± 0.45	0.475	0.635
Anxiety	1.63 ± 0.42	1.67 ± 0.36	0.716	0.475
Hyperactivity index	2.55 ± 0.56	2.53 ± 0.57	0.281	0.779

PSQ, conners parent symptom questionnaire. The results in the table were the average scores of all items in each domain.

Conduct scores were significantly lower in the MNF-CBT group (1.74 ± 0.35) than in the MNF group (1.89 ± 0.47) (*t* *=* 2.590, *P* *=* 0.010) (Table 4). Similarly, learning scores improved more in the MNF-CBT group (1.66 ± 0.44) compared to the MNF group (1.83 ± 0.42) (*t* *=* 2.798, *P* *=* 0.006). Psychosomatic disorders scores decreased significantly in the MNF-CBT group, with a score of 1.02 ± 0.36 compared to 1.16 ± 0.38 in the MNF group (*t* *=* 2.733, *P* *=* 0.007). The MNF-CBT group also showed a greater reduction in impulsivity and hyperactivity scores (1.73 ± 0.41) relative to the MNF group (1.84 ± 0.31) (*t* *=* 2.177, *P* *=* 0.031). Anxiety scores were significantly lower in the MNF-CBT group (1.32 ± 0.32) vs. the MNF group (1.44 ± 0.36) (*t* *=* 2.644, *P* *=* 0.009). Lastly, the hyperactivity index was significantly reduced in the MNF-CBT group (1.94 ± 0.37) when compared with the MNF group (2.13 ± 0.63) (*t* *=* 2.853, *P* *=* 0.005). These results suggest that the addition of CBT to the nutritional formula leads to more pronounced changes in behavior and symptom measures in children with ADHD.

**Table 4:** Comparison of PSQ score between two groups (after treatment).

Parameters	MNF group (*n* = 112)	MNF-CBT group (*n* = 108)	*t*	*P*
Conduct	1.89 ± 0.47	1.74 ± 0.35	2.590	0.010
Learning	1.83 ± 0.42	1.66 ± 0.44	2.798	0.006
Psychosomatic disorders	1.16 ± 0.38	1.02 ± 0.36	2.733	0.007
Impulsivity and hyperactivity	1.84 ± 0.31	1.73 ± 0.41	2.177	0.031
Anxiety	1.44 ± 0.36	1.32 ± 0.32	2.644	0.009
Hyperactivity index	2.13 ± 0.63	1.94 ± 0.37	2.853	0.005

Inattention (IA) scores were similar, with the MNF group scoring 27.83 ± 3.53 and the MNF-CBT group scoring 28.15 ± 3.42 (*t* *=* 0.679, *P* *=* 0.498) (Table 5). Hyperactivity/impulsivity (HI) scores were 14.78 ± 3.25 for the MNF group and 14.57 ± 3.31 for the MNF-CBT group (*t* *=* 0.469, *P* *=* 0.640). Lastly, oppositional (OP) scores were comparable between the MNF group at 14.02 ± 3.16 and the MNF-CBT group at 13.89 ± 3.21 (*t* *=* 0.303, *P* *=* 0.762). These similar baseline scores indicate that the groups were well-matched prior to the initiation of treatment.

**Table 5:** Comparison of SNAP-IV-teacher form score between two groups (before treatment).

Parameters	MNF group (*n* = 112)	MNF-CBT group (*n* = 108)	*t*	*P*
IA	27.83 ± 3.53	28.15 ± 3.42	0.679	0.498
HI	14.78 ± 3.25	14.57 ± 3.31	0.469	0.640
OP	14.02 ± 3.16	13.89 ± 3.21	0.303	0.762

SNAP-IV, Swanson, Nolan, and Pelham-IV; IA, inattention; HI, hyperactivity/impulsivity; OP, oppositional. The results in the table were the sum of all items in each domain.

**Figure 1:**
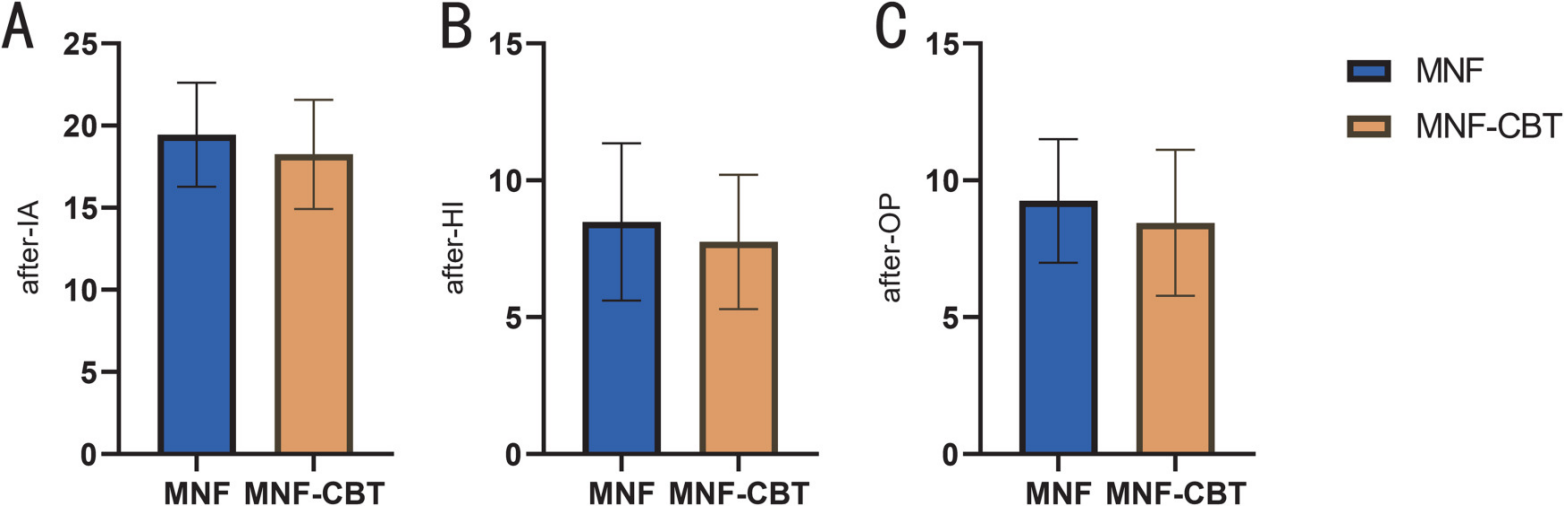
Comparison of SNAP-IV-teacher form score between two groups (after treatment). **(A)** IA; **(B)** HI; **(C)** OP. SNAP-IV, Swanson, Nolan, and Pelham-IV; IA, inattention; HI, hyperactivity/impulsivity; OP, oppositional. The results in the table were the sum of all items in each domain. *: *P* < 0.05; **: *P* < 0.01.

Inattention (IA) scores were significantly lower in the MNF-CBT group, with an average score of 18.25 ± 3.33 compared to 19.45 ± 3.17 in the MNF group (*t* *=* 2.735, *P* *=* 0.007) (Figure 1). Similarly, hyperactivity/impulsivity (HI) scores showed a greater reduction in the MNF-CBT group, scoring 7.75 ± 2.46 vs. 8.48 ± 2.87 in the MNF group (*t* *=* 2.027, *P* *=* 0.044). The oppositional (OP) scores were also significantly improved, with the MNF-CBT group achieving a mean of 8.45 ± 2.67, compared to 9.25 ± 2.26 in the MNF group (*t* *=* 2.418, *P* *=* 0.016). These findings suggest that the integration of CBT with the nutritional formula may enhance symptom management in children with ADHD.

### 3.3 Functional impairment

Prior to the treatment, there were no significant differences in D-DTODS scores between the MNF group and the MNF-CBT group (*P* > 0.05) (Table 6). After treatment, prior to school, the MNF-CBT group scored significantly lower (1.74 ± 0.58) compared to the MNF group (1.93 ± 0.63) (*t* *=* 2.26, *P* *=* 0.025) (Table 7). In-school scores also favored the MNF-CBT group, with scores of 1.77 ± 0.43 compared to 1.98 ± 0.66 in the MNF group (*t* *=* 2.790, *P* *=* 0.006). Similarly, after school, the MNF-CBT group had a lower average score (1.57 ± 0.51) compared to the MNF group (1.73 ± 0.56) (*t* *=* 2.158, *P* *=* 0.032). Evening scores were significantly reduced in the MNF-CBT group (2.69 ± 0.69) relative to the MNF group (2.93 ± 0.75) (*t* *=* 2.476, *P* *=* 0.014). Finally, before-bed scores were notably lower in the MNF-CBT group (1.66 ± 0.52) vs. the MNF group (1.89 ± 0.64) (*t* *=* 3.004, *P* *=* 0.003). These results suggest that the combination of CBT with the nutritional formula was more effective at mitigating difficult times throughout the day for children with ADHD. To further elucidate the clinical relevance of our findings, Cohen's d effect sizes were calculated for all continuous variables comparisons and are reported in Table 8.

**Table 6:** Comparison of D-DTODS score between two groups (before treatment).

Parameters	MNF group (*n* = 112)	MNF-CBT group (*n* = 108)	*t*	*P*
Before school (items 1–3)	2.45 ± 0.47	2.41 ± 0.52	0.532	0.595
In school (items 4–6)	2.75 ± 0.53	2.62 ± 0.54	1.880	0.061
After school (item 7)	2.23 ± 0.61	2.27 ± 0.68	0.484	0.629
Evening (item 8)	3.42 ± 0.24	3.38 ± 0.21	1.484	0.139
Before bed (items 9 & 10)	2.55 ± 0.37	2.62 ± 0.41	1.491	0.137

D-DTODS, Dundee difficult times of the day scale. The results in the table were the average scores of all items in each domain.

**Table 7:** Comparison of D-DTODS score between two groups (after treatment).

Parameters	MNF group (*n* = 112)	MNF-CBT group (*n* = 108)	*t*	*P*
Before school (items 1–3)	1.93 ± 0.63	1.74 ± 0.58	2.26	0.025
In school (items 4–6)	1.98 ± 0.66	1.77 ± 0.43	2.790	0.006
After school (item 7)	1.73 ± 0.56	1.57 ± 0.51	2.158	0.032
Evening (item 8)	2.93 ± 0.75	2.69 ± 0.69	2.476	0.014
Before bed (items 9 & 10)	1.89 ± 0.64	1.66 ± 0.52	3.004	0.003

**Table 8:** Cohen's d effect sizes for comparisons.

Parameters	Cohen_d
Age (years)	0.065
BMI (kg/m²)	0.137
Duration of illness (year)	0.190
Daily medication (mg)	0.070
(before) Conduct	0.199
(before) Learning	0.183
(before) Psychosomatic disorders	0.170
(before) Impulsivity and hyperactivity	0.064
(before) Anxiety	0.097
(before) Hyperactivity index	0.038
(after) Conduct	0.348
(after) Learning	0.377
(after) Psychosomatic disorders	0.369
(after) Impulsivity and hyperactivity	0.295
(after) Anxiety	0.357
(after) Hyperactivity index	0.381
(before) IA	0.092
(before) HI	0.063
(before) OP	0.041
(after) IA	0.369
(after) HI	0.273
(after) OP	0.326
(before) Before school (items 1–3)	0.072
(before) In school (items 4–6)	0.254
(before) After school (item 7)	0.065
(before) Evening (item 8)	0.200
(before) Before bed (items 9 & 10)	0.201
(after) Before school (items 1–3)	0.305
(after) In school (items 4–6)	0.373
(after) After school (item 7)	0.291
(after) Evening (item 8)	0.334
(after) Before bed (items 9 & 10)	0.404

## 4 Discussion

In this study, we investigated the impact of a combined intervention of a MNF and CBT in managing symptoms of children with ADHD.

One plausible explanation for the observed impact of the MNF-CBT combination relates to the synergistic effects of nutrition and psychological intervention on neurodevelopmental processes. Micronutrients were essential for brain function—they play critical roles in synaptic plasticity, neurotransmitter synthesis, and overall neuronal health (20). Deficiencies in elements such as zinc, magnesium, iron, and vitamin B complex have been linked to behavioral. Zinc, for instance, was known to modulate the glutamatergic system, which was implicated in the pathophysiology of ADHD. Magnesium's involvement in controlling NMDA receptors further underscores the role of these micronutrients in neuroprotection and modulation of excitatory neurotransmitters. The supplementation of these essential nutrients may rectify underlying biochemical imbalances, thus creating a physiologically conducive environment for effective psychotherapy (21–23).

Simultaneously, CBT directly addresses maladaptive thought patterns and behaviors, fostering cognitive restructuring and emotional regulation. The skills taught in CBT—cognitive restructuring, emotion regulation techniques, social skills training, and family involvement—provide a framework for individuals to understand and manage their symptoms better. This multidimensional approach enhances not only the ability of the children to handle stress and anxiety but also significantly impacts attention regulation mechanisms. The regular structure, plus the strategies for coping and adjustment provided by CBT, likely complement the neurobiological benefits of the MNF, thereby amplifying the overall therapeutic effect (24–26).

The significantly reduced scores in conduct problems, learning difficulties, and psychosomatic disorders within the MNF-CBT group as compared to the MNF group can be attributed to this dual-action mechanism. While MNF might be addressing the neurochemical deficits, CBT targets behavioral and emotional dysregulation, providing children with practical tools to apply to everyday challenges and reducing the stress associated with academic and social interactions. In a typical day for a child with ADHD, having effective coping strategies could alleviate perceived obstacles, thus reducing scores on scales measuring difficult times of the day (27, 28).

Furthermore, the significant reduction in inattention, hyperactivity/impulsivity, and oppositional symptoms within the MNF-CBT group suggests an enhanced capacity for self-regulation. This change was particularly important in a school setting, where the demand for sustained attention and appropriate behavior was high. The structured environment component of CBT, including consistent routines and phased task completion with rewards, likely plays a crucial role in cultivating these improvements. Children learn to anticipate and manage daily demands better, contributing to lower scores in inattention and hyperactivity post-treatment (29, 30). Our findings align with those of Pan et al. (31), who demonstrated that combining CBT with medication in adults with ADHD led to significant improvements in core ADHD symptoms, depression, and psychological quality of life. Unlike Pan et al.'s focus on medicated adults, our study shows that a combined intervention of MNF and CBT can achieve similar outcomes in children, addressing both neurochemical deficits and cognitive-behavioral factors. This suggests that the synergistic effects of MNF and CBT may provide a more comprehensive approach for managing ADHD symptoms in pediatric populations, potentially offering broader benefits than either intervention alone.

From a broader perspective, these findings have important implications for the management of ADHD in children. The traditional approach often favors pharmacological interventions aimed primarily at symptom suppression, with limited attention to underlying nutritional deficiencies or cognitive strategies (32). Our study suggests that a more holistic approach, combining dietary supplements with CBT, might offer a sustainable model of care by addressing both the symptomatic and root-cause dimensions of these disorders.

Additionally, the results underline the necessity of personalized treatment plans. Given the diversity in biological and social factors contributing to ADHD, an integrated treatment plan tailored to the individual child's needs might yield more consistent and replicable outcomes. This approach would account for the individual variance in response to micronutrient supplementation and cognitive therapies. It is important to consider the cost and resource requirements of implementing such interventions at scale. The need for specialized dietary planning and regular therapy sessions could pose significant barriers in terms of both financial and human resources. Future research should explore strategies to make these interventions more cost-effective and feasible in real-world settings.

The study, however, does have limitations that warrant further exploration. The retrospective nature of this study limits the ability to establish causality between the interventions and outcomes. The non-randomized assignment of participants based on CBT availability introduces potential selection bias, which may affect the interpretation of the results. The lack of blinding for outcome assessors may introduce bias, particularly in subjective measures, further impacting the validity of the findings. The exclusion of children with comorbidities such as intellectual disabilities and severe physical illnesses may limit the generalizability of our findings to the broader ADHD population.

One of the strengths of our study is the standardized dietary control for the MNF group. But maintaining such strict dietary control in real-world settings may be challenging due to individual preferences, cultural differences, and practical limitations, which could affect feasibility and compliance. Both groups received MNF, with no group receiving CBT only or standard care alone. This design makes it impossible to isolate the independent effect of CBT. Therefore, caution should be exercised when interpreting the results, as the observed improvements cannot be definitively attributed solely to CBT without considering its interaction with MNF. Future studies employing a randomized controlled trial design with more complete control arms, such as a group receiving only CBT and another receiving standard care, could address this issue and provide clearer insights into the independent effects of each intervention. The intervention period and follow-up duration were relatively short, thus long-term impact and stability of the combined MNF-CBT intervention remain to be evaluated. Future studies should aim to examine the persistency of these improvements over extended periods and investigate the potential of declining benefits if the interventions were ceased. We did not conduct subgroup analyses to explore whether specific groups (e.g., age, gender, ADHD subtype) benefited more from these interventions. Future studies should address this gap to better understand how different populations respond to these treatments. While this study focuses on a specific age group, it would be beneficial to explore if similar approaches can effectively address adult ADHD symptoms, recognizing that the neurodevelopmental needs evolve with age.

Moreover, while our study was robust in assessing multiple outcome measures, exploring additional biomarkers and neuroimaging studies could provide insight into the underlying physiological changes induced by the MNF and CBT combination. Understanding how these interventions structurally and functionally alter brain circuits associated with attention and impulse control could unlock new pathways for diagnosis and treatment.

## 5 Conclusion

In conclusion, the integration of MNF with CBT emerges as a promising approach for children with ADHD. This dual approach not only mitigates symptoms more effectively but potentially enhances overall quality of life by fostering better interpersonal relationships and academic achievements. Building on these findings through further research could lead to significant advances in the personalized treatment strategies for neurodevelopmental.

## Data Availability

The original contributions presented in the study are included in the article/Supplementary Material, further inquiries can be directed to the corresponding author.
